# Costs Related to Diverting Ileostomy After Rectal Cancer Surgery: A Population-Based Healthcare Cost Analysis Based on Nationwide Registers

**DOI:** 10.1177/00469580231212126

**Published:** 2023-12-17

**Authors:** Ursula Dahlstrand, Pontus Gustafsson, Pia Näsvall, Jeaneth Johansson, Ulf Gunnarsson, Ulrik Lindforss

**Affiliations:** 1 Karolinska Institutet, Stockholm, Sweden; 2 Enköping Hospital, Enköping, Sweden; 3 Visby Hospital, Visby, Sweden; 4 Umeå University, Umeå, Sweden; 5 Luleå University of Technology, Luleå, Sweden; 6 Halmstad University, Halmstad, Sweden; 7 Karolinska University Hospital, Stockholm, Sweden

**Keywords:** defunctioning stoma, stoma reversal, low anterior resection, socioeconomic factors, cost analysis, healthcare costs, rectal neoplasms, ileostomy, resource allocation, inpatients

## Abstract

Low anterior resection for rectal cancer often includes a diverting loop-ileostomy to avoid the severe consequences of anastomotic leakage. Reversal of the stoma is often delayed, which can incur health-care costs on different levels. The aim is to, on population basis, determine stoma-related costs, and to investigate habitual and socioeconomic factors associated to the level of cost. Multi-register design with data from the Swedish Rectal Cancer Registry, the National Prescribed Drug Register, Statistics Sweden and cost-administrative data from the National Board of Health and Welfare. Data was gathered for 3564 patients with rectal cancer surgery 2007 to 2013, for 3 years following the surgery. Factors influencing the cost of inpatient care and stoma-related consumables were assessed with linear regression analyses. All monthly costs were higher for females (consumables *P* < .001 and in-patient care *P* = .031). Post-secondary education (*P* = .003) and younger age (*P* = .020) was associated with a higher cost for consumables while suffering a surgical complication was associated with increased cost for inpatient care (*P* < .001). Patients who had their stoma longer had lower monthly costs (consumables *P* < .001 and in-patient care *P* < .001). Female gender, longer duration of stoma, young age, and higher education are associated with higher costs for the care of a diverting stoma after rectal cancer surgery. This study does not allow for analyses of causality but the results together with deepened analyses of underlying reasons form a proper basis for decisions in health care planning and allocation of resources. These findings may have implications on the debate of equal care for all.

**What do we already know about this topic?**
Healthcare resources in Europe are ever decreasing. This is the first study to determine and compare the costs of delaying stoma reversal after cancer surgery.
**How does your research contribute to the field?**
This three-year follow-up study estimates the costs of stoma-related inpatient care as well as disposables used in stoma care after rectal cancer surgery.
**What are your research’s implications toward theory, practice, or policy?**
The findings of this large population-based study should improve future surgical decision-making and planning and allocation of healthcare resources.

## Introduction

Colorectal cancer is the third most common cancer in men and second in women, and it is the third most deadly malignant disease in the world.^1^ In Sweden, with a population of about 10 million, approximately 2100 new cases of adenocarcinoma of the rectum are diagnosed each year (incidence 0.021%).^2^ The risk of anastomotic leakage after rectal cancer surgery is relatively high and several studies^3-5^ have shown that creation of a diverting stoma in low anastomosis rectal cancer surgery substantially improves the outcome of leakage despite similar leakage rates.^6^

Care of a diverting stoma is expensive for the healthcare system and society, but this should be balanced against the high cost of advanced care should anastomotic leakage occur without diversion. The total cost of stoma care is reduced by reversal of the stoma as soon as is surgically possible. In a time of dwindling healthcare resources, allocation of resources has become more pragmatic, where surgical treatment of patients with a malignant disease is given priority over those with a benign condition. Unfortunately, the surgical literature provides little information regarding the costs of care of a diverting stoma and its delayed reversal.

Traditionally, a diverting loop-ileostomy is reversed 3 months after surgery or a month or so after completing adjuvant chemotherapy. Delayed reversal of a diverting stoma is common^7^ and 20% eventually become permanent.^7,8^ Early reversal of a diverting loop-ileostomy has been investigated in small trials that have shown better quality-of-life and lower costs.^9-11^ Common reasons for delaying reversal are adjuvant chemotherapy, surgical complications including anastomotic leakage, or limited resources such as shortage of staff or when key resources required are unavailable due to other operations being given priority.^7,12^ Despite the considerable consequences of delayed reversal of or permanent diverting stomas for the healthcare system and society, no population-based studies have addressed this question. Better knowledge about the socioeconomic consequences of delayed stoma reversal is essential to plan and allocate resources effectively, from both the patient’s and healthcare system’s perspective. By shedding some light on these consequences, resources may be used in a better way; essential in a situation where shortage of resources is a growing problem even in wealthy countries.

The aim of this population-based study was to determine the costs of stoma care in rectal cancer patients with a diverting stoma, and to investigate habitual and socioeconomic factors that influence the level of these costs.

## Methods

Eligible patients were identified by requesting data from the Swedish Colon and Rectal Cancer Register (SCRCR) regarding all patients who underwent anterior resection with a diverting stoma for rectal cancer between 1st January 2007 and 31st December 2013. The study is of explorative and descriptive character. Approximately 500 rectal cancer surgeries with a diverting stoma are performed annually, and a seven-year sample containing some 3500 patients from the national population-based register was chosen to assure a reliable description of the relationships between steps in the health-care chain and health economics.

The SCRCR is a nationwide Swedish rectal cancer quality register established in 1995 with 98%-99% coverage.^13,14^ It includes information regarding patient characteristics, preoperative work-up, procedural details, tumor characteristics, postoperative complications, planned oncological therapy, and follow-up results.

Socioeconomic data regarding level of education and disposable income at the time of cancer surgery for all eligible patients were obtained from Statistics Sweden, the administrative agency responsible for producing and distributing government and official statistics.

The study period for each patient included began with date of cancer surgery and ended 3 years after, or at the time of stoma reversal or death. Patients emigrating or dying prior to end of follow-up were censored. Costs associated with the diverting stoma were calculated using data from administration registers kept by the National Board of Health and Welfare.

Cost of consumables (drugs and stoma care disposable products) related to the stoma were acquired from the National Prescribed Drug Register (NPDR), which contains data regarding all dispensed prescribed drugs and medical disposables, such as ostomy appliances.^15^ All pharmacies, retailers, and wholesalers are obliged to report their sales to the Swedish Health Agency, which in turn submits information to the NDPR. Data on all drugs and appliances dispensed are registered together with the patient’s personal identity number.^16^ In addition to information about the patient, the NPDR includes information about the product prescribed (ATC code or equivalent), the prescription dispensed (quantity, dates prescribed and dispensed, type of prescription), the costs (total cost, patient cost, cost carried by the welfare system), and the prescriber. All data on prescriptions for stoma care disposables or anti-propulsive drugs used to regulate stoma output (ATC code A07DA) from the date of stoma creation (date of cancer surgery) and 3 years on, were obtained for those patients included.

Hospital admissions during the study period for causes related to the stoma were identified through the National Patient Register (NPR).^17^ The NPR has 99% coverage and registers data on patient (national personal identity number), geographical (hospital, county council, *etc*), administrative (type of care and date of admission and discharge), and medical (diagnoses and procedures). A Diagnosis-Related Group (DRG) code according to the NordDRG system used in Scandinavia is assigned to every observation in the NPR. It is mandatory for all county councils and healthcare providers to report inpatient care information to the NPR. For the patients included, all records containing a diagnosis related to high stoma output or dysfunction of the stoma (ICD-10 codes K52.9A, K91.4, Z46.5, Z43.2, Z93.2, E86.9, or N99.0) or a surgical procedure related to the stoma during the study period, were acquired.

The primary outcome was costs per month of stoma-related disposables and stoma-related inpatient care. The actual costs for consumables in Swedish Crowns (SEK) included those paid for by the patient and those covered by the healthcare authority. To account for possible effects caused by inflation, the cost for each dispensation was inflation adjusted to the cost it would have corresponded to in January 2022. The tool for adjustment was provided by Statistics Sweden and is based on Consumer Price Index, the standard measure of compensation and inflation calculations in Sweden. The cost for inpatient care was calculated by multiplying the DRG code for each visit with 63 493 SEK (the value assigned to 1 DRG according to NordDRG in 2022)^18^ and taking the sum of costs of all admissions for each patient during the study period. The reason for using the assigned value of 2022 for all visits, rather than using the assigned value for the year of the specific visit was to avoid possible inflation associated effects. The sums were converted to Euros (EUR) according to the average exchange rate for 2022 (1 EUR = 10.6349 SEK).

**Figure 1.**
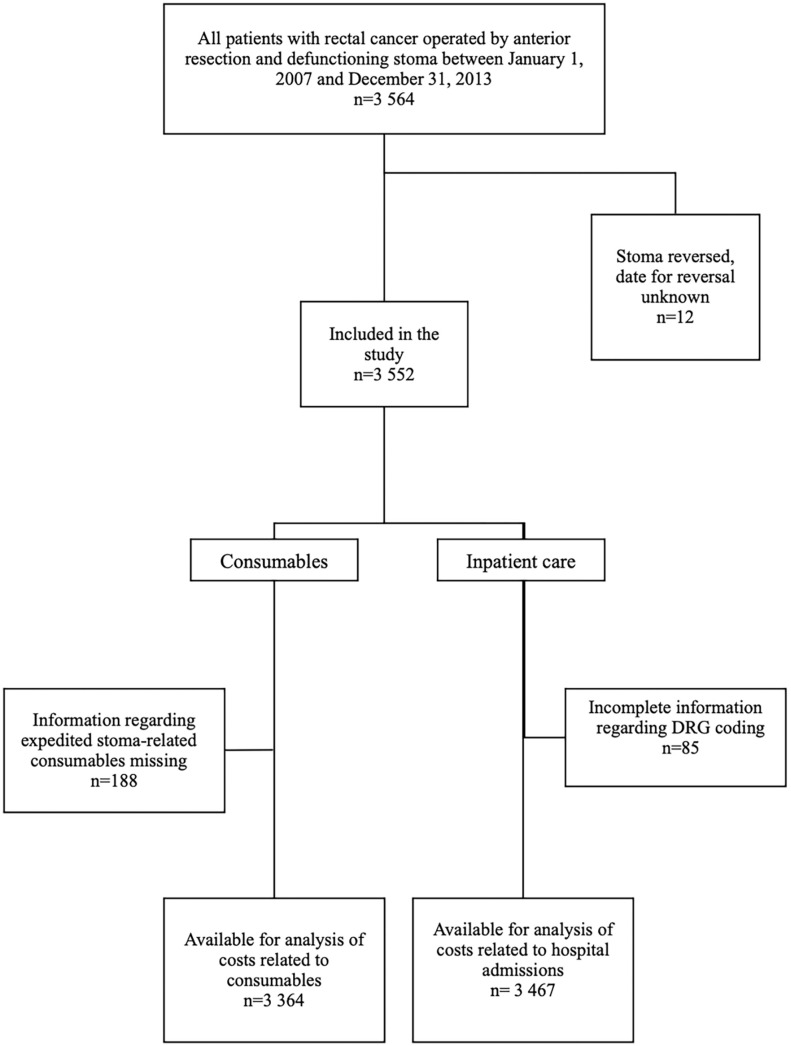
Flow-chart over included patients with low anterior resection and a diverting loop-ileostomy in 2007 to 2013.

Data for 3564 patients were extracted from the registers, 12 patients were excluded since it was not possible to establish when their stoma was reversed, leaving 3552 for final inclusion in the study (Figure 1).

This study was approved by the Regional Ethics Review Board of Stockholm (EPN Dnr 2018/538-32). Patients in Sweden have the possibility to opt-out from participation in national quality registers. For those who choose not to opt-out, written informed consent is not considered required for each register study and was thus waived by the Ethics Review Board.

### Statistical Analyses

Statistical analyses were performed using Stata/SE 16.1 (StataCorp, College Station, TX, USA). Central trend and dispersion of monthly costs was described using the median and the interquartile range (IQR) or range. Linear regression analyses were performed to detect factors associated with costs of diverting stoma per month. Factors assessed were age, gender, postoperative complication (within 30 days of cancer surgery), planned adjuvant chemotherapy, disposable income in EUR, acquired level of education (lower secondary/upper secondary/postsecondary education), cancer stage according to the Union for International Cancer Control (UICC), American Society of Anesthesiologists (ASA) physical status class, and time with stoma (months). Determining factors for the multivariable analyses were selected based on presumed relevance. Separate analyses were conducted for costs for consumables and costs related to inpatient care. In the analysis regarding cost for consumables, the 2.5% of patients with the highest expense and the 2.5% of patients with the lowest expense were excluded to avoid influence of extreme values. For inpatient care no such exclusion was made since the DRG system does not report actual cost per patient but rather expected cost for a patient with the diagnoses reported. List wise deletion was used for cases with missing data.

## Results

The final study cohort comprised 3552 patients with a diverting stoma after anterior resection of the rectum due to rectal cancer, 2173 (61.2%) were male and 1379 (38.8%) were female. Characteristics are reported in Table 1. The median age at the time of the index operation and the creation of the loop-ileostomy was 66 years (range 23-90 years). Most patients (58.2%) had ASA Class 2, only 7% had Stage IV cancer, while Stages I, II, and III were roughly equal in occurrence (28%, 28%, and 34%, respectively). Approximately one-third of patients were planned for adjuvant chemotherapy after cancer surgery. The highest level of education was postsecondary in 38.4%, upper secondary in 38.4%, and lower secondary in 28.2% of patients. Disposable income, inflation adjusted, had an IQR between 14 401 and 26 928 EUR/year (median 18 933 EUR/year). Patients had their stoma, that is time from stoma creation to reversal, end of study, or death, for a median of 7.1 months (IQR 4.3-11.3 months).

**Table 1.** Patient Characteristics and Stoma Reversal Data for 3552 Patients Who Had Rectal Cancer Surgery with a Diverting Loop-Ileostomy 2007 to 2013.

Median age, years (range)	66 (23-90)
Sex: no. (%)	
Female	1379 (38.8)
Male	2173 (61.2)
Physical status/ASA Class: no. (%)	
1	895 (25.2)
2	2070 (58.2)
3-4	530 (14.9)
Missing	57 (1.6)
Cancer stage: no. (%)	
0-1	996 (28.0)
2	993 (28.0)
3	1223 (34.4)
4	248 (7.0)
Missing	92 (2.6)
Planned adjuvant chemotherapy: no (%)	
Yes	1209 (34.0)
No	2281 (64.2)
Missing	62 (1.8)
Level of education: no. (%)	
Lower secondary	1002 (28.2)
Upper secondary	1365 (38.4)
Postsecondary	880 (24.8)
Missing	305 (8.6)
Median disposable income, EUR (IQR)	18 933 (14 401-26 928)
Median time with stoma, months (IQR)	7.1 (4.2-11.3)

The median inflation adjusted monthly cost for stoma-related consumables was 155.7 EUR (range 10.8-438.5 EUR when extreme values were excluded, 0.2-1272.8 EUR if they were included), information regarding cost of consumables was missing for 188 patients in the NPDR. The median inflation adjusted total cost for supplies during the duration of the stoma was 1120.1 EUR (range 11.4-15 508.3 EUR if extreme values were excluded, range 5.2-17 975.3 EUR if extreme values were included). Inpatient care due to stoma-related medical issues had a median cost per month of 1502.5 EUR in (IQR 882.3-2480.5 EUR). The median total cost for inpatient stoma-related care during the duration of the stoma was 92 064.9 EUR (IQR 88 255.3-137 779.8 EUR). There were 421 patients who were never admitted for stoma-related inpatient care between cancer surgery and stoma reversal, end of study, or death.

The multivariable linear regression analysis regarding monthly cost for consumables (Table 2) showed an association between female gender and higher cost (coefficient for males –40.92; 95% CI –47.17 to –34.68; *P* < .001). Monthly costs were also higher for younger patients (age coefficient –0.47; 95% CI –0.80 to –0.13; *P* = .006) and patients with postsecondary education (coefficient 11.68; 95% CI 3.42 to 19.94; *P* = .006). Patients with ASA Class 3 or 4 had higher monthly costs than patients with ASA 1 (coefficient 12.41; 95% CI 2.12 to 22.69; *P* = .018). Patients who had their diverting stoma for a longer time had lower monthly costs for consumables (coefficient –1.26; 95% CI –1.61 to –0.91; *P* < .001).

**Table 2.** Multivariable Linear Regression for Monthly Cost in EUR for Stoma-Related Consumables (Stoma Dressings and Medication Regulating Bowel Function) for 2826 Patients With a Deviating Stoma After Anterior Resection Due to Rectal Cancer.

Number of observations: 2826				95% Confidence interval	
	Coef	Std. err	*P*-value	Min	Max
Age (years)	−0.47	0.17	.006	−0.80	−0.13
Sex					
Female	1 (ref)				
Male	−40.92	3.19	<.001	−47.17	−34.68
Postoperative complication					
No	1 (ref)				
Yes	−1.14	3.19	.72	−7.39	5.12
Adjuvant chemotherapy					
No	1 (ref)				
Yes	−3.10	4.12	.45	−11.17	4.97
Disposable income (1000 EUR)	0.092	0.067	.17	−0.040	0.22
Lower secondary education	1 (ref)				
Upper secondary education	3.59	3.67	.45	−3.61	10.80
Postsecondary education	11.68	4.21	.006	3.42	19.94
Cancer stage I	1 (ref)				
Cancer stage II	0.35	4.00	.93	−7.49	8.20
Cancer stage III	1.72	4.60	.71	−7.30	10.74
Cancer stage IV	0.59	6.89	.93	−12.92	14.09
ASA 1	1 (ref)				
ASA 2	2.29	3.72	.54	−5.01	9.59
ASA 3-4	12.41	5.25	.018	2.12	22.69
Time with stoma (months)	−1.26	0.18	<.001	−1.61	−0.91

Regarding costs for stoma-related inpatient care (Table 3), multivariable linear regression analysis revealed higher monthly costs for patients who had a postoperative complication after index surgery (coefficient 1683.58; 95% CI 989.39 to 2377.76; *P* < .001) and for females (male coefficient –757.65; 95% CI –1451.55 to –63.75; *P* = .031), while those who had their diverting stoma for a longer time had lower hospital costs per month (coefficient –325.57; 95% CI –374.45 to –276.68; *P* < .001).

**Table 3.** Multivariable Linear Regression for Cost per Month for Inpatient Care due to Stoma-Related Medical Issues for 2804 Patients With a Diverting Stoma After Anterior Resection for Rectal Cancer.

Number of observations: 2804				95% Confidence interval	
	Coef	Std. err	*P*-value	Min	Max
Age (years)	1.87	18.76	.92	−34.90	38.65
Sex					
Female	1 (ref)				
Male	−757.65	353.88	.032	−1451.55	−63.75
Postoperative complication					
No	1 (ref)				
Yes	1683.58	354.03	<.001	989.39	2377.76
Adjuvant chemotherapy					
No	1 (ref)				
Yes	−82.22	464.35	.86	−992.73	828.29
Disposable income (1000 EUR)	−4.27	9.09	.64	−22.09	13.55
Lower secondary education	1 (ref)				
Upper secondary education	−244.58	409.37	.55	−1047.28	558.12
Postsecondary education	−138.44	470.63	.77	−1061.26	784.39
Cancer stage I	1 (ref)				
Cancer stage II	166.05	443.50	.71	−703.57	1035.67
Cancer stage III	745.98	512.75	.15	−259.43	1751.39
Cancer stage IV	4084.73	812.92	<.001	2490.75	5678.71
ASA 1	1 (ref)				
ASA 2	518.78	409.96	.21	−285.08	1322.64
ASA 3-4	241.06	589.29	.68	−914.42	1396.55
Time with stoma (months)	−325.57	24.93	<.001	−374.45	−276.68

## Discussion

In this nationwide population-based study, female gender was associated with higher costs for consumables and stoma-related inpatient care compared to men. This relationship was also evident in a multivariable linear regression model compensating for other influencing factors. Although the present data do not allow for causal analysis of the reason for this relationship, it could be that this was due to greater consideration to the specific requirements of women, especially esthetic aspects. Patient wishes and attitudes among healthcare staff can influence the choice of disposable products as well as the intensity of caring for the diverting stoma. Similar gender differences have also been described in end-stage care of advanced colorectal cancer.^19^ Other possible reasons include differences in body contour and distribution of subcutaneous fat,^20^ as well as connective tissue properties including the proportion of elastin.^21^

Being well-educated and young was also associated with higher costs at both the individual, healthcare system and societal level. Most younger people are part of the workforce and often carry out physically demanding activities necessitating more frequent changes of stoma disposables and, in some cases, requiring more expensive products. Well-educated patients may have higher demands regarding comfort and functionality of stoma disposables which could also be a contributing factor to the higher monthly costs in the younger group. In a recent trial, higher level of education and young age were associated with fewer psychosocial problems among stoma patients.^22^ This might partly be explained by a more active approach against the caregivers among these groups of patients. Other interesting aspects not covered in this paper are the attitudes of healthcare workers and the influence of expectations and perceptions among stoma nurses and other care providers. These should be the subject of future qualitative studies.

Another interesting finding was the fact that monthly costs seemed not to be associated with stage of cancer (apart from higher inpatient care cost for patients with Stage IV cancer) nor with adjuvant chemotherapy. This means that the side-effects of chemotherapy, given in most Stage III cases and possible treatment of disseminated disease in Stage IV cases, do not significantly influence the costs of a diverting stoma. This is in contrast to a recent Australian study where neoadjuvant therapy was associated with increased cost of delayed reversal of the stoma despite times to reversal being similar to ours.^23^ One reason for these conflicting results may be that the considerably larger number of patients included in the present population-based trial allowed for adjustments for other influencing factors.

Complications within 30 days of index surgery often leads to delayed discharge or readmission which is known to affect total healthcare costs,^24^ as was the case in the present study where costs for inpatient care during the study period (3 years after index surgery) were higher in the group with complications. However, this did not apply to stoma-related disposables. One reason could be that costs were calculated for the entire period, diluting any increased costs over a short period after the index operation or later admission for a surgical complication. This hypothetically increased cost seems to be of limited overall importance since it is not apparent in the mean costs for the entire period studied. From the life-long perspective, however, an anastomotic leak would still lead to increased total costs for stoma care and consumables since this is one of the most important risk factors for a diverting stoma becoming permanent.^25^

The median time with a stoma was 7.1 months (from creation to reversal, end of study, or death). It is obvious that time to reversal is strongly associated with a higher total cost for stoma care, at the individual level as well as for the health care system and society. The present study was conducted before the pandemic era, and our cost estimates can thus be used to calculate the cost burden of delayed stoma reversal due to lack of available resources in routine care.

Non-reversal of the stoma during the three-year follow-up was seen in 478 patients (13.5%), and it is highly probable that many of those diverting stomas will become permanent. This is also congruent with a recent single-center study on frequency and risk factors for non-reversal of a diverting stoma.^25^ Having a diverting stoma formed from the distal part of the small intestine is not without risk for complication such as high output volume leading to dehydration and possibly renal failure, a well-known problem among the elderly.^26,27^ High stoma output and dehydration after ileostomy formation has in a recent meta-analysis been shown to be the leading reason for readmission, incurring considerable health-care costs.^28^ Other problems associated with a diverting stoma are peristomal dermatitis and ulceration, parastomal hernia, and skin irritation.^27^ It may well be that with time these complaints increase in frequency and dignity causing even more suffering and expense. These negative effects have during recent years initiated an ongoing discussion regarding a more selective use of diverting stomas.

A strength of the present study is that it is based on data from nationwide well-established and validated registers. This assures external validity and generalizability of our results. A previous study from the Netherlands revealed that care provider data is more valid in terms of consumption of medication and care products than patient-reported estimates.^29^ Furthermore, the study model included several aspects ranging from habitual factors and surgical parameters, to need for healthcare and cost of stoma disposables and medication, which gives a rather complete description of cost to the healthcare system and to society.

A weakness is the lack of dynamic data on costs during the study period. Costs likely varied during the period, such as higher costs for inpatient care and stoma disposables during the months after the index operation, and that such a dynamic effect would be seen as a higher cost per month for cases with a shorter time to stoma reversal. Another weakness is the lack of data for costs to society including sick leave; an aspect that warrants further research. Lastly, as with all epidemiological studies based on register data, analyses are limited to the variables included in the register and do not allow for dependent causal analysis of relevant occurrences.

## Conclusion

Female gender, younger age, and higher level of education were associated with higher costs for care of a diverting stoma after rectal cancer surgery. The present study did not allow for analyses of causality, but we believe our results can form the basis for better surgical decision-making and planning and allocation of healthcare resources. Research is needed on how to make healthcare more equal, according to the specific needs of the patient, as well as the avoidance of decisions based on stereotype factors such as gender, age, and education.

## References

[1] Ferlay J Shin HR Bray F , et al. Estimates of worldwide burden of cancer in 2008: GLOBOCAN 2008. Int J Cancer. 2010;127(12):2893-2917. doi:10.1002/ijc.25516 21351269

[2] Norr R . Rektalcancer 2021 - Nationell kvalitetsrapport för år 2021 från Svenska Koloncancerregistret. 2022. Accessed April 4, 2023, https://cancercentrum.se/globalassets/cancerdiagnoser/tjock–och-andtarm-anal/kvalitetsregister/tjock–och-andtarm-2022/rektalrapport2021_2022-05-24.pdf

[3] Matthiessen P Hallbook O Rutegard J Simert G Sjodahl R. Defunctioning stoma reduces symptomatic anastomotic leakage after low anterior resection of the rectum for cancer: a randomized multicenter trial. Ann Surg. 2007;246(2):207-214. doi:10.1097/SLA.0b013e3180603024 17667498 PMC1933561

[bibr4-00469580231212126] Montedori A Cirocchi R Farinella E Sciannameo F Abraha I. Covering ileo- or colostomy in anterior resection for rectal carcinoma. Cochrane Database Syst Rev. 2010;12(5);CD006878. doi:10.1002/14651858.CD006878.pub2 PMC12721704 20464746

[5] Bertelsen CA Andreasen AH Jørgensen T Harling H . Danish Colorectal Cancer Group. Anastomotic leakage after anterior resection for rectal cancer: risk factors. Colorectal Dis. 2010;12(1):37-43. doi:10.1111/j.1463-1318.2008.01711.x 19175624

[6] Snijders HS van den Broek CB Wouters MW , et al. An increasing use of defunctioning stomas after low anterior resection for rectal cancer. Is this the way to go? Eur J Surg Oncol. 2013;39(7):715-720. doi:10.1016/j.ejso.2013.03.025 23632318

[7] Floodeen H Lindgren R Matthiessen P. When are defunctioning stomas in rectal cancer surgery really reversed? Results from a population-based single center experience. Scand J Surg. 2013;102(4):246-250. doi:10.1177/1457496913489086 24056133

[8] Kuryba AJ Scott NA Hill J van der Meulen JH Walker K. Determinants of stoma reversal in rectal cancer patients who had an anterior resection between 2009 and 2012 in the English National Health Service. Colorectal Dis. 2016;18(6):O199-O205. doi:10.1111/codi.13339 27005316

[9] Danielsen AK Park J Jansen JE , et al. Early closure of a temporary ileostomy in patients with rectal cancer: a multicenter randomized controlled trial. Ann Surg. 2017;265(2):284-290. doi:10.1097/SLA.0000000000001829 27322187

[bibr10-00469580231212126] Park J Angenete E Bock D , et al. Cost analysis in a randomized trial of early closure of a temporary ileostomy after rectal resection for cancer (EASY trial). Surg Endosc. 2020;34(1):69-76. doi:10.1007/s00464-019-06732-y 30911920 PMC6946724

[11] Park J Danielsen AK Angenete E , et al. Quality of life in a randomized trial of early closure of temporary ileostomy after rectal resection for cancer (EASY trial). Br J Surg. 2018;105(3):244-251. doi:10.1002/bjs.10680 29168881 PMC5814870

[12] Gustafsson CP Gunnarsson U Dahlstrand U Lindforss U. Loop-ileostomy reversal-patient-related characteristics influencing time to closure. Int J Colorectal Dis. 2018;33(5):593-600. doi:10.1007/s00384-018-2994-x 29508050 PMC5899111

[13] Moberger P Sköldberg F Birgisson H. Evaluation of the Swedish Colorectal Cancer Registry: an overview of completeness, timeliness, comparability and validity. Acta Oncol. 2018;57(12):1611-1621. doi:10.1080/0284186X.2018.1529425 30477372

[14] Norr R . Rektalcancer - Nationell kvalitetsrapport för år 2016 från Svenska Kolorektalcancerregistret. 2017. Accessed April 4, 2023, https://cancercentrum.se/globalassets/cancerdiagnoser/tjock–och-andtarm-anal/kvalitetsregister/rapporter-2017/rektal2016.pdf

[15] Socialstyrelsen. National Prescribed Drug Register. 2020. Accessed June 20, 2023, https://www.socialstyrelsen.se/en/statistics-and-data/registers/national-prescribed-drug-register/

[16] Ludvigsson JF Otterblad-Olausson P Pettersson BU Ekbom A. The Swedish personal identity number: possibilities and pitfalls in healthcare and medical research. Eur J Epidemiol. 2009;24(11):659-667. doi:10.1007/s10654-009-9350-y 19504049 PMC2773709

[17] Socialstyrelsen. National Patient Register. 2019. Accessed June 20, 2023, https://www.socialstyrelsen.se/en/statistics-and-data/registers/national-patient-register/

[18] Socialstyrelsen (National Board of Health and Welfare). Viktlistor för NordDRG. 2019. Accessed May 20, 2023, https://www.socialstyrelsen.se/statistik-och-data/klassifikationer-och-koder/drg/viktlistor/

[19] Mittmann N Liu N Porter JM , et al. End-of-life home care utilization and costs in patients with advanced colorectal cancer. J Community Support Oncol. 2014;12(3):92-98. doi:10.12788/jcso.0025 24971414

[20] Han J Liu X Tang M , et al. Abdominal fat and muscle distributions in different stages of colorectal cancer. BMC Cancer. 2023;23(1):279. doi:10.1186/s12885-023-10736-2 PMC10044362 36978044

[21] Ayadh M Guillermin A Abellan MA Bigouret A Zahouani H. The assessment of natural human skin tension orientation and its variation according to age for two body areas: forearm and thigh. J Mech Behav Biomed Mater. 2023;141:1-10. doi:10.1016/j.jmbbm.2023.105798 36996528

[22] Dellafiore F Manara DF Arrigoni C , et al. Predictors of adjustment to living with an ostomy: results of a cross-sectional study. Adv Skin Wound Care. 2022;35(5):1-6. doi:10.1097/01.ASW.0000823980.15166.35 35442922

[23] Barnard J Milne T Teo K , et al. Causes and costs of delayed closure of ileostomies in rectal cancer patients in Australasian units. ANZ J Surg. 2023;93(3):636-642. doi:10.1111/ans.18092 36203387

[24] Zheng V Wee IJY Abdullah HR , et al. Same-day discharge (SDD) vs standard enhanced recovery after surgery (ERAS) protocols for major colorectal surgery: a systematic review. Int J Colorectal Dis. 2023;38(1):110. doi:10.1007/s00384-023-04408-7 37121985 PMC10149457

[25] Thomas F Menahem B Lebreton G , et al. Permanent stoma after sphincter preservation for rectal cancer. a situation that occurs more often than you might think. Front Oncol. 2022;12:1-9. doi:10.3389/fonc.2022.1056314 PMC9909408 36776358

[26] Gessler B Haglind E Angenete E. A temporary loop ileostomy affects renal function. Int J Colorectal Dis. 2014;29(9):1131-1135. doi:10.1007/s00384-014-1949-0 25026894

[27] Åkesson O Syk I Lindmark G Buchwald P. Morbidity related to defunctioning loop ileostomy in low anterior resection. Int J Colorectal Dis. 2012;27(12):1619-1623. doi:10.1007/s00384-012-1490-y 22576906

[28] Vogel I Shinkwin M van der Storm SL , et al. Overall readmissions and readmissions related to dehydration after creation of an ileostomy: a systematic review and meta-analysis. Tech Coloproctol. 2022;26(5):333-349. doi:10.1007/s10151-022-02580-6 35192122 PMC9018644

[29] van den Brink M van den Hout WB Stiggelbout AM van de Velde CJ Kievit J. Cost measurement in economic evaluations of health care: whom to ask? Med Care. 2004;42(8):740-746. doi:10.1097/01.mlr.0000132351.78009.a1 15258475

